# The expression of Delta ligands in the sponge *Amphimedon queenslandica* suggests an ancient role for Notch signaling in metazoan development

**DOI:** 10.1186/2041-9139-3-15

**Published:** 2012-07-23

**Authors:** Gemma S Richards, Bernard M Degnan

**Affiliations:** 1 School of Biological Sciences, University of Queensland, Brisbane, QLD, 4072, Australia

## Abstract

**Background:**

Intercellular signaling via the Notch pathway regulates cell fate, patterning, differentiation and proliferation, and is essential for the proper development of bilaterians and cnidarians. To investigate the origins of the Notch pathway, we are studying its deployment in a representative of an early branching lineage, the poriferan *Amphimedon queenslandica*. The *A. queenslandica* genome encodes a single Notch receptor and five membrane-bound Delta ligands, as well as orthologs of many genes that enact and regulate canonical Notch signaling events in other animals.

**Methods:**

In the present report we analyze the structure of the five *A. queenslandica* Deltas using bioinformatic methods, and characterize their developmental expression via whole mount *in situ* hybridization and histological staining.

**Results:**

Sequence analysis of the *A. queenslandica* Delta ligands highlights the conservation of their extracellular domains. This contrasts with the divergence of their intracellular regions, each of which is predicted to bear a unique repertoire of protein interaction motifs. In keeping with this diversity, these ligands are expressed differentially and dynamically throughout *A. queenslandica* embryogenesis, both in cell type specific patterns and broader regional domains. Notably, this expression coincides with the development of the photosensitive larval pigment ring, the non-ciliated cuboidal cells located at the anterior pole of the larva, and the intraepithelial flask cells and globular cells that are presumed to have sensory and/or secretory roles.

**Conclusions:**

Based on the dynamic and complex patterns of expression of these Delta ligands and the Notch receptor, we propose that the Notch signaling pathway is involved in regulating the development of diverse cell types in *A. queenslandica*. From these observations we infer that Notch signaling is a conserved feature of metazoan development, ancestrally contributing to cell determination, patterning and differentiation processes.

## Background

Intercellular signaling pathways drive animal development by facilitating cellular communication and the coordination of morphogenetic processes. Of the major developmental signaling pathways, comparative studies have revealed that core components of the Wnt, Notch, transforming growth factor β (TGFβ) and receptor tyrosine kinase (RTK) pathways are encoded in the genomes of representative species from all major extant animal clades [1,2]. Here, we focus on the evolution of one of these pathways, the Notch signaling pathway, which provides a mechanism for short-range, localized signaling between directly apposing cells (reviewed in [3-5]).

At the molecular level, a Notch signaling event is initiated by the binding of a Delta/Serrate type ligand to the Notch receptor. In response, the receptor undergoes a series of proteolytic cleavages that results in the production of a short intracellular signaling fragment, the Notch intracellular domain (NICD). The NICD then translocates to the nucleus of the receiving cell, where it binds to the CBF1/Suppressor of hairless/Lag1 (CSL) repressor complex and elicits a change in the transcriptional activity of the cell. Commonly, Notch signaling events act to regulate the responsiveness of individual cells, or cell populations, to the developmental instructions they encounter [6]. In this capacity, Notch signaling has been shown to direct cell specification, differentiation and proliferation, delineate boundaries between developmental fields, and regulate cell migration and apoptotic events (reviewed in [3,7]).

Broad-ranging examples of Notch pathway activity have been described in bilaterians, and recent studies in the Cnidaria have implicated Notch signaling in the differentiation of the interstitial cell lineage and boundary formation in the hydrozoan *Hydra*, and in nervous system development of the anthozoan *Nematostella vectensis*[8-11]. These reports of canonical Notch signaling in Cnidaria indicate that the eumetazoan ancestor also likely deployed the Notch pathway in a range of developmental processes.

Here, we analyze the sequence and developmental expression of Delta and Notch genes in the demosponge *Amphimedon queenslandica* and thereby contribute to the understanding of the role of this signaling pathway in earlier branching metazoan clades, that is, Porifera, Ctenophora and *Trichoplax*[12,13]. Previously, we and others have reported that the genome of *A. queenslandica* encodes the molecular components of the canonical Notch pathway, including ligands, a receptor and CSL transcription factor, as well as many genes with a role in Notch activation, regulation and inhibition [14,15]. We have also described the expression of the *A. queenslandica* Notch receptor and a single ligand, *AmqDelta1*, during the ontogeny of the globular cell lineage in late embryonic stages [14]. In this report, we expand our analysis by documenting the expression of the *Notch* receptor and five *Delta* ligands across *A. queenslandica* embryogenesis. We describe the molecular structure of the five *Delta* genes in detail, and discuss the possible functional significance of their diversification. In addition, we present histological sections to describe *A. queenslandica* development in greater detail, and better contextualize the gene expression patterns. This study reveals a highly dynamic and complex pattern of *Notch* and *Delta* expression, consistent with Notch signaling playing a role in morphogenesis and cell fate determination in this demosponge.

## Results

### Sequence analysis of *A. queenslandica* Deltas

**Figure 1:**
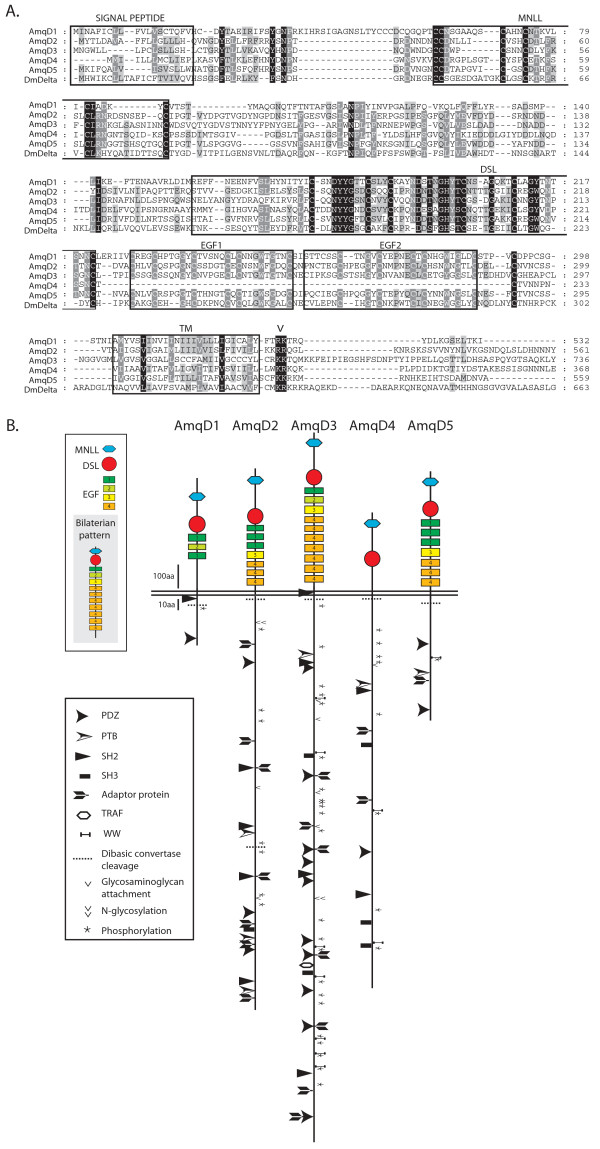
**Molecular characteristics of *Amphimedon queenslandica *Deltas. **(**A**) Alignment of the extracellular regions of *A. queenslandica* and *Drosophila* Deltas. All sequences possess a signal peptide and transmembrane (TM) domain, a Delta/Serrate/Lag (DSL) domain, epidermal growth factor (EGF) repeats (except AmqD4) and the conserved pattern of cysteine residues characteristic of the MNLL region. A series of basic residues (V) lies downstream of the TM domain, possibly representing a nuclear localization sequence. The region between EGF2 and the TM domain is omitted. Dashes indicate gaps, residues are shaded according to the level of conservation at each position: 100%, black; 80%, dark grey; 60%, light grey. (**B**) The diversification of the *A. queenslandica* Delta ligands is reflected in the number and organization of their extracellular EGF domains, and the distribution and identity of predicted interaction sites in their intracellular tails. EGF repeat identities and bilaterian consensus pattern follow [18]. Potential sites of protein binding, cleavage, phosphorylation and glycosyl modification based on [19]. PTB, phosphotyrosine-binding domain; SH2, Src homology 2; SH3, Src homology 3; TRAF, tumor necrosis factor receptor-associated factor.

An alignment of the *A. queenslandica* Deltas highlights their conserved regions (Figure 1A, Additional file 1). All sequences possess two hydrophobic regions (a signal peptide and a transmembrane domain) characteristic of single pass transmembrane (TM) proteins. Following the TM domains is a series of basic residues that may function as a nuclear localization sequence (Figure 1A), and all AmqDeltas also possess valines located several residues N-terminal to the end of the TM domain, which can be possible sites for γ-secretase cleavage [16]. Next to the signal peptide in bilaterian Deltas is a MNLL domain. When using domain recognition software this domain is only detected in AmqDelta4, however all AmqDeltas possess the conserved pattern of cysteine residues characteristic of the MNLL region (Figure 1A). A Delta/Serrate/Lag (DSL) domain, which mediates binding to Notch receptors in bilaterians, is also present in all five proteins. Downstream of the DSL domain, all proteins, with the exception of AmqDelta4, have a series of epidermal growth factor (EGF) repeats ranging in number from three (AmqDelta1) to ten (AmqDelta3) (Figure 1B). AmqDelta2, AmqDelta3 and AmqDelta5 have EGF repeats containing predicted sites for modification by *O*-fucosyltransferase and Fringe (based on [17]).

The phylogenetic relationships between *A. queenslandica* Delta ligands and other metazoan Notch ligands have been analyzed elsewhere [15]. Briefly, the *A. queenslandica* Deltas group with all other metazoan Deltas to the exclusion of all Jagged type ligands [15]. AmqDelta1, 2, 4 and 5 form a monophyletic clade, whereas AmqDelta3 lies within a poorly resolved clade, which also includes sequences from *LottiaStrongylocentrotus* and *Helobdella*[15]. As poor resolution of DSL phylogenies is common, it has been suggested that analysis of the number and spacing of the cysteine residues in the EGF repeat domains of the ligands can be informative about their relationships [18]. In this way, EGF repeats are annotated 1 to 4 on the basis of their cysteine arrangements, and an ancestral EGF organization in Delta proteins has been proposed for the Bilateria (Figure 1B) [18]. AmqDelta3 has an EGF repeat pattern most similar to that of bilaterians, suggesting that it has retained a more ancestral domain arrangement while the other ligands have diverged from this organization (Figure 1B).

All ligands are predicted to possess a variety of functional linear motifs in their intracellular tails (ICT) (Figure 1B). All contain multiple lysines in their ICTs, as well potential phosphorylation sites, although only AmqDeltas2 to 4 also have residues that could be sites for glycosaminoglycan attachments, and only AmqDeltas2 and 3 have sites that may be *N*-glycosylated. All ligands also contain PDZ domain binding sites, and all except AmqDelta1 have sites that may interact with the adaptor protein complex. Several ligands also possess WW domain binding motifs, and a number of Src homology 2 (SH2) and phosphotyrosine-binding (PTB) domain binding sites are proposed in AmqDeltas1 to 4 and AmqDeltas2 to 5 respectively. Src Homology 3 (SH3) domain binding sites are predicted in AmqDeltas2 to 4 and a tumor necrosis factor receptor associated factor (TRAF) binding site is predicted in AmqDelta3 only. These data should be interpreted with the caveat that hits returned by ELM are not assigned significance and are only intended to act as a guide to sites of interest [19]. Nonetheless, it appears that each of the five AmqDelta proteins possesses a unique repertoire of interaction sites in its intracellular domain.

### Developmental expression of the *A. queenslandica* Notch receptor and ligands

#### *AmqDelta1*

**Figure 2:**
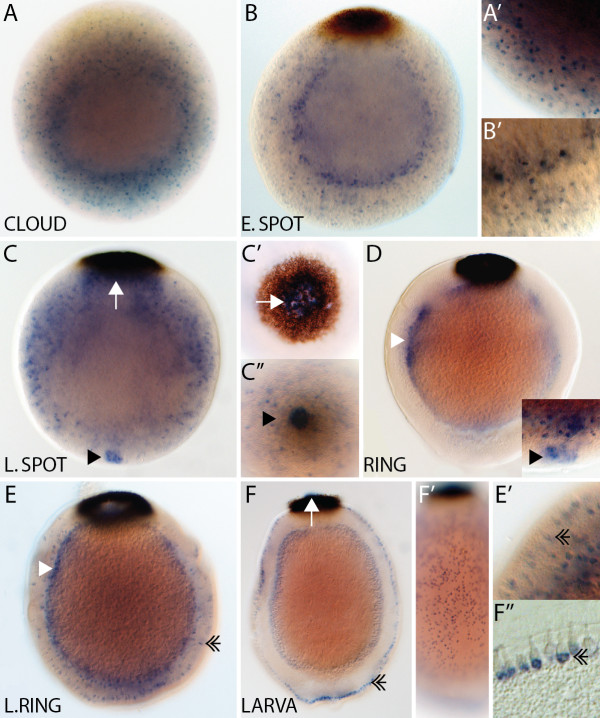
***AmqDelta1 *developmental expression. **(**A**) Expression of *AmqDelta1* is first detected in cells in the outer layer in early cloud stage embryos (**A’**). (**B**) Expression remains in scattered cells (**B’**) in the outer layer, more densely occurring at the boundary between the two layers. (**C**) Late spot stage embryos express *AmqDelta1* in the outer layer, as well as in cells under the center of the forming pigment ring (white arrow) (**C’**) and at the anterior pole (arrowhead) (**C”**). (**D**) Ring stage embryos display expression in the forming middle layer (white arrowhead), as well as the anterior pole (inset). (**E**) In late ring embryos, expression is detected in the subepithelial (middle) layer (white arrowhead), and in the globular cells (double arrowhead) that are migrating from the subepithelial layer to the outer margin (**E’**). (**F**) Expression in larvae persists in the globular cells (double arrowhead), now located around the outer margin (**F’,F”**), and within the pigment ring (white arrow). All panels display cleared, whole mount embryos, except (F”) in which the embryo was sectioned after staining. (C”), anterior view; (C’), posterior view; all remaining panels are lateral views with posterior to the top.

*AmqDelta1* transcripts are not detected by *in situ* hybridization during cleavage (data not shown), with this gene first being notably expressed in the early cloud stage in scattered cells predominantly located in the outer layer (Figure 2A,A’). This expression persists into the early spot stage, with some cells coalescing around the boundary between the two forming cell layers (Figure 2B,B’). At spot stage, a cluster of cells at the anterior pole is detected, and expression in this area is maintained through to the ring stage (Figure 2C,C”,D). *AmqDelta1* is also expressed in the center of the forming pigment ring at the late spot stage (Figure 2C,C’). Middle (subepithelial) layer expression is apparent in the ring (Figure 2D) and late ring stage (Figure 2E), and in the population of globular cells that migrate from the middle to the outer layer (Figure 2E’), as previously observed [14]. In the larva, expression of *AmqDelta1* is predominantly in the globular cells located amongst the cells of the outer layer, as well as inside the pigment ring (Figure 2F,F”) [14].

#### * AmqDelta2 *

**Figure 3:**
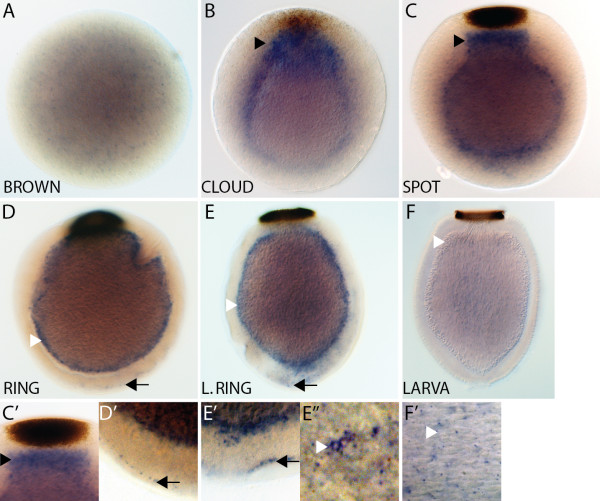
***AmqDelta2 *developmental expression. **(**A**) Brown stage, isolated cells express *AmqDelta2* in no discernable pattern. (**B**) At late cloud stage, expression is under the forming pigment spot (arrowhead) at the posterior pole. (**C**) Spot stage embryos show strong expression of *AmqDelta2* under the spot (arrowhead) (**C’**), as well as some expression at the boundary between the middle and outer layers. (**D**) Ring stage embryos no longer display an expression domain under the posterior pole, expression is now localized to the forming subepithelial layer (white arrowhead) and to a small population of cells at the anterior margin of the embryo (arrow) (D). (**E**) In late ring stages, staining persists in the anterior margin (arrow) (**E’**) and subepithelial layer (white arrowhead) (**E”**) and is apparent in the inner cell mass. (**F**) Little expression is detected in the larva, except for some cells in the subepithelial layer (white arrowhead) (**F’**). All panels display cleared whole mount embryos, lateral views with posterior to the top.

Detection of* AmqDelta2 *expression commences at late cleavage (Figure 3A), in a small number of scattered cells. In the cloud stage, expression is highest in a group of cells that are located beneath and distinct from the forming pigment spot at the posterior of the embryo (Figure 3B), and this expression is maintained through to the spot stage (Figure 3C,C’). In ring embryos, *AmqDelta2* is expressed in the forming middle layer, as well as in the flask cells, located around the anterior third of the embryo (Figure 3D,D’). Flask cells continue to express *AmqDelta2* in the late ring stage; transcripts are also present in the middle and inner layers (Figure 3E,E”). In the larva, transcripts are localized to the cells of the subepithelial layer (Figure 3F,F’).

#### * AmqDelta3 *

**Figure 4:**
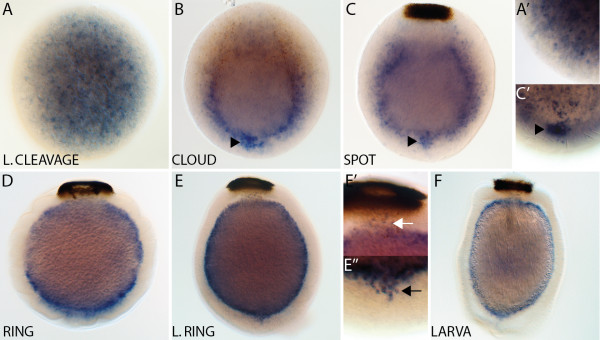
***AmqDelta3 *developmental expression. **(**A**) Expression commences during late cleavage in cells scattered throughout the embryo (**A’**). (**B**) As pigment cells begin to migrate to the posterior pole, expression of *AmqDelta3* is in cells found at the boundary between the two forming cell layers, including a group of cells located towards the anterior of the embryo (arrowhead). (**C**) Similar to the cloud stage, expression in spot embryos is in cells located around the boundary between cell layers, with an anterior condensation of expression (arrowhead) ((**C’**) note: this image is from an early spot stage embryo, not the embryo pictured in (C)). (**D**) At the early ring stage, expression has coalesced in the forming middle layer. (**E**) Expression in the late ring stage is localized to the subepithelial layer, as well as a small number of cells located within the outer layer at the anterior (arrow) and posterior (white arrow) poles (**E’,E”**). (**F**) Expression in the larval stage remains in the subepithelial layer. All panels display cleared whole mount embryos. (C’), anterior view; all remaining panels are lateral views with posterior to the top.

* AmqDelta3 *expression is first detected at late cleavage in cells scattered throughout the embryo (Figure 4A,A’). In the cloud stage, transcripts are detected in cells around the forming boundary between the inner and outer cell layers, and there is a noticeable cluster of *AmqDelta3*-expressing cells at the anterior of the boundary region (Figure 4B). This cluster persists through to the spot stage (Figure 4C,C’). In ring (Figure 4D) and late ring (Figure 4E,E”) embryos, expression is localized to the forming subepithelial layer, and this persists into the larval stage (Figure 4F).

#### * AmqDelta4 *

**Figure 5:**
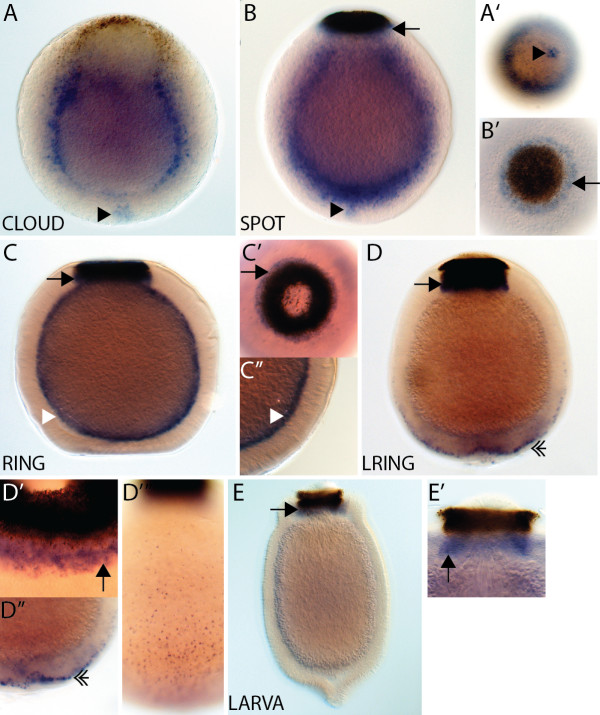
***AmqDelta4 *developmental expression. **(**A**) At cloud stage, *AmqDelta4* expression is in cells located near the boundary between the inner and outer layers of the embryo, as well as in a patch of cells that lie beneath the anterior pole (arrowhead) (**A’**). (**B**) At spot stage, expression persists in cells at the boundary between the inner and outer layers, and beneath the anterior pole (arrowhead), and a new expression domain arises around the outer edge of the pigment spot (arrow) (**B’**). (**C**) Ring stage expression is strong under the forming ring (arrow) (**C’**) and in the middle layer (white arrowhead) (**C”**). (**D**) Late ring embryos show no expression in the subepithelial layer; expression under the ring remains strong (arrow) (**D’**), and expression arises in cells located at the outer margin of the embryo, more densely occurring in the anterior third (double arrowhead) (**D”,D”’**). (**E**) Larval expression is predominantly under the pigment ring (arrow) **(E”)**. All panels display cleared whole mount embryos. (A’), anterior view; (B’,C’,D’), posterior views; all remaining panels are lateral views with posterior to the top.

* AmqDelta4 *is first detected by *in situ* hybridization in cells at the boundary of the forming cell layers in cloud stage embryos, as well as in a cluster of cells at the anterior pole (Figure 5A,A’). These anterior cells continue to express *AmqDelta4* until the late spot stage (Figure 5B; data not shown). A domain of expression is also evident around the pigment spot at this stage (Figure 5B’), which later narrows in ring stage embryos (Figure 5C,C’) and then is located underneath the pigment ring in later embryos (Figure 5D,D’) and larvae (Figure 5E,E’). In ring embryos, the forming middle layer strongly expresses *AmqDelta4* (Figure 5C,C”); in late ring embryos, *AmqDelta4* is also expressed in the flask cell population, around the anterior third of the embryo (Figure 5D,D”,D”’).

#### * AmqDelta5 *

**Figure 6:**
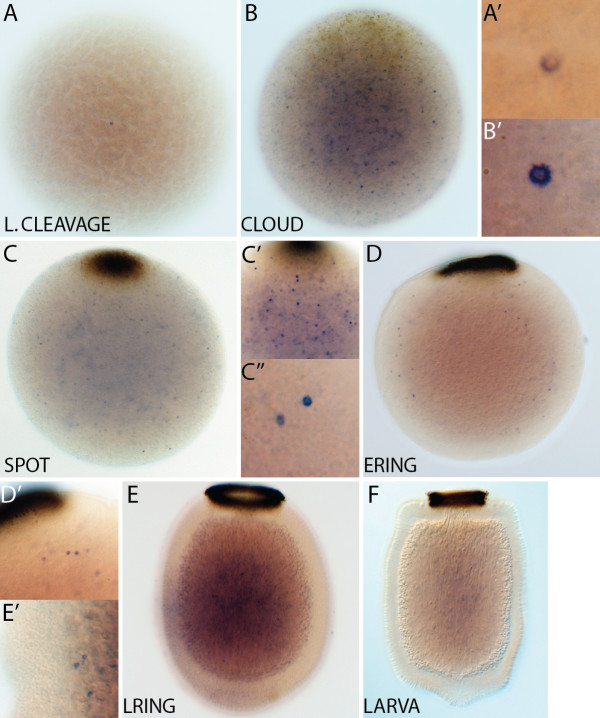
***AmqDelta5 *developmental expression.** Across all developmental stages, *AmqDelta5* is expressed in a small number of spatially isolated cells throughout the embryo. These are scattered through all layers in earlier stages (**A**-**C**), becoming more localized to the developing middle and inner layers in later stages (**D**-**E**). Fewer cells are apparent in the ring and larval stages (**E**,**F**). All panels display cleared whole mount embryos, lateral views with posterior to the top.

Throughout development,* AmqDelta5 *is expressed in scattered, isolated cells that are located throughout all cell layers of the embryos (Figure 6). Fewer cells are apparent in later stages, where they appear to be more restricted to the middle layer and inner cell mass.

#### *AmqNotch*

**Figure 7:**
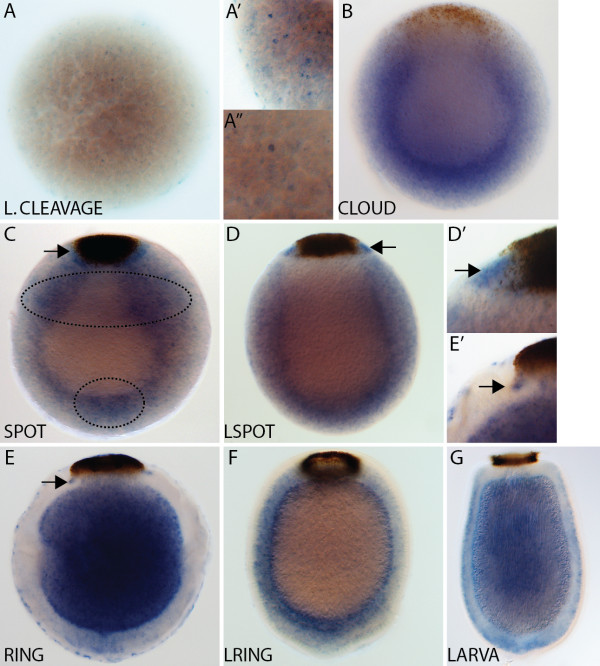
***AmqNotch *developmental expression. **(**A**) In late cleavage stages, expression is detected in scattered cells (**A’,A”**). (**B**) Cloud stage, expression is diffuse throughout the embryo, with denser staining at the boundary between the inner and outer layers. (**C**) Expression remains diffuse throughout the spot stage embryo, although is somewhat concentrated in two areas (circled); a domain of higher expression is noted around the pigment spot (arrow). (**D**) Expression persists into the late spot stage, a domain of higher expression remains localized to cells surrounding the pigment spot (arrow) (**D’**). (**E**) In the ring stage, expression of *AmqNotch* has coalesced to the forming middle layer as well as remaining associated with the pigment ring (arrow) (**E’**); expression also is detected in the inner cell mass. (**F**) Expression in late ring embryos is within the subepithelial layer and outer layer. (**G**) *AmqNotch* is expressed throughout the larva. (Note: expression around the outer margin of the ring embryo (E) is in cells that make up the non-embryonic follicle layer.) All panels display cleared whole mount embryos, lateral views with posterior to the top.

During late cleavage *AmqNotch* expression is detected in cells distributed throughout the embryo (Figure 7A-A”). By cloud stage, expression is diffuse, but predominantly in the outer layer (Figure 7B). This general expression is maintained in the spot stage, with higher levels of expression detected around the pigment spot, as well as at the anterior and posterior of the boundary between the inner and outer cell layers (Figure 7C). *AmqNotch* expression is broad and diffuse in late spot embryos, with a domain of higher expression around the forming pigment ring (Figure 7D,D’). In ring embryos, expression is no longer evident in the outer cell layer, instead, transcripts are detected in the inner cell mass, as well as surrounding the forming pigment ring (Figure 7E,E’). Expression in late ring embryos is in the middle and outer layers (Figure 7F); in the larva *AmqNotch* is weakly expressed throughout (Figure 7G).

### Cytological context of *A. queenslandica* Notch/Delta expression

**Figure 8:**
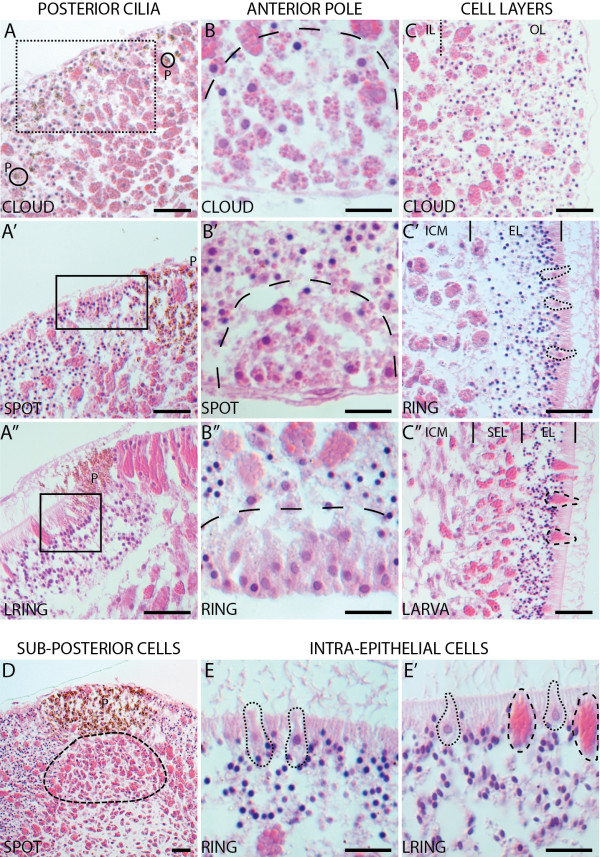
**Cytological features of *Amphimedon queenslandica *development.** All panels show sectioned embryos stained with hematoxylin and eosin. (**A-A”**) Posterior ciliated cells. Cloud, no cell population uniquely lies in the future position of the posterior ciliated cells, dotted box (A). Spot, cells lateral to the pigment spot are morphologically distinct, box (A’). Late ring, these cells have apparently ingressed, and appear to be clustered, box (A”). (**B-B”**) Anterior pole. Cloud, distinct cells with small inclusions are present at the anterior pole, dashed line (B). Spot, the anterior-most cells are condensing at the pole, dashed line (B’). Ring, the anterior pole contains a group of non-ciliated cuboidal cells with apical nuclei, dashed line (B”). (**C-C”**) Cell layers. Cloud, the inner layer contains large granular macromeres, the outer layer contains micromeres and large globular macromeres (C). Ring, the outer layer contains epithelial cells, interspersed with flask cells, the inner layer is comprised of large macromeres among other cell types (C’). The larva has three cells layers; an outer epithelial layer interspersed with globular cells and flask cells, a subepithelial layer composed mostly of large macromeres, and the inner cell mass (C”). (**D**) Subposterior region. Inner layer macromeres are more densely packed directly under the forming pigment spot. Dashed line, approximates the area of higher cell density. (**E,E’**) Intraepithelial cells. Ring, the epithelial layer contains flask cells, dotted lines (E). Larva, both flask cells and globular cells (dashed lines) are present (**E’**). P, pigment cells; IL, inner layer; ICM, inner cell mass; OL, outer layer; EL, epithelial layer; SEL, subepithelial layer.

Using hematoxylin and eosin we stained sectioned *A. queenslandica* embryos to identify morphological features that corresponded to prominent areas of Notch/Delta expression during development (Figure 8). Regions highlighted by gene expression include the posterior ciliated cells (which together with the pigment cells comprise the pigment ring), the cuboidal cells of the anterior pole, the subepithelial (middle) layer, a region under the forming pigment spot and the intraepithelial flask and globular cells (Figure 8A-E, respectively).

Prior to pigment spot formation, there is no distinctive cell population found specifically at the future position of the posterior ciliated cells (Figure 8A). By spot stage, cells just anteriolateral to the pigment spot are distinguished from the surrounding epithelial cells due to their columnar morphology and alignment (Figure 8A’), coincident with the spatial expression of *AmqNotch* and *AmqDelta4* at this stage (Figures 5B and 7B). In late ring embryos, these cells have further elongated internally, and are polarized; nuclei are basal, cilia are apical and the cells are in small clusters (Figure 8A”). The expression of *AmqNotch* and *AmqDelta4* is now also seen in a narrower domain that lies just below the surface of the embryo (Figures 5D,D’ and 7E’). The larval expression of *AmqDelta4* remains in the region under the pigment ring, the presumptive location of the nuclei of the cells bearing the long posterior cilia (Figure 5E).

At the anterior pole of the *A. queenslandica* larva is a cluster of cuboidal non-ciliated cells [20]. At cloud stage, a distinct cell population is already visible in the vicinity of the anterior pole (Figure 8B), at a time in which *AmqDelta4* expression is noted in this region, while *AmqDelta3* is located to the interior (Figures 4B and 5A). By spot stage, a group of cells, morphologically similar to those seen in the area during the cloud stage, are condensing into a cluster at the anterior pole (Figure 8B’), both *AmqDelta1* and *AmqDelta4* are expressed at this pole (Figures 2C,C” and 5B). In the ring stage embryo, the anterior pole is clearly distinguished from the surrounding ciliated epithelial cells (Figure 8B”); *AmqDelta1* is still expressed at the anterior pole (Figure 2D).

The *A. queenslandica* embryo is initially organized into two layers during development from brown to spot stages; a third layer becomes evident in the transition from spot to larva. At the cloud/spot stage, the inner layer is comprised of large granular macromeres while the outer layer contains both micromeres and large globular macromeres (Figure 8C). Most of the ligands are expressed in patterns that would suggest their localization to cells belonging to the large macromere population, predominantly in the outer layer, and around the boundary between the two layers (Figures 2A,B, 3B,C, 4B,C and 5A,B). By the ring stage, the outer layer contains no macromeres, being comprised only of epithelial cells, interspersed with flask cells (Figure 8C’). The inner layer consists of a mixture of large macromeres and various unidentified cell types (Figure 8C’). At this point in development, all genes except *AmqDelta5* are localized to the region in which the middle layer is forming (Figures 2D, 3D, 4D, 5C and 7E). In the larva, three cells layers are evident: an outer epithelial layer interspersed with globular cells and flask cells; a subepithelial layer, composed mostly of large macromeres running in a perpendicular orientation to the anterior-posterior axis; and an inner cell mass in which spicule-containing sclerocytes and amoeboid cell types are embedded in a dense collagenous matrix (Figure 8C”). *AmqDelta3* remains expressed in the larval subepithelial layer (Figure 4F).

Uniquely, *AmqDelta2* is strongly expressed directly under the forming pigment spot (Figure 3B,C). A distinct cell population is not noted in this region, however in cloud (not shown) and spot stage embryos, the inner layer macromeres are more densely packed in this area (Figure 8D).

The larval epithelial layer is composed of ciliated columnar cells interspersed with flask cells and globular cells [20]. Flask cells appear amongst the anterior third of the epithelial layer in ring and late ring embryos (Figure 8E), coincident with *AmqDelta2* and *4* expression in isolated cells around the anterior periphery (Figures 3D and 5D). In the larva, (Figure 8E’), *AmqDelta1* and *4* are expressed in patterns that suggest they are localized to the globular cells and flask cells respectively (Figures 2F and 5E). The globular cells, expressing *AmqDelta1*, are found around the entire larva and appear to migrate to this position from the subepithelial layer during the ring stage, as previously described (Figure 2E) [14].

## Discussion

Here, we describe the structure and expression patterns of five Delta ligands and the Notch receptor throughout the embryonic development of the demosponge *Amphimedon queenslandica*, building upon our previous analysis of *AmqNotch* and *AmqDelta1* in the ontogeny of one larval cell type, the globular cell [14]. The more detailed and comprehensive characterization of Notch and Delta expression in this current study reveals a likely contribution of the Notch signaling pathway to the orchestration of a number of aspects of *A. queenslandica* development.

### Ligand function and evolution

It is probable that there was a single Delta-type ligand in the metazoan last common ancestor (LCA), with the Jagged/Serrate-type ligands being added to the pathway in the stem lineage of the Cnidaria + Bilateria [15]. The independent diversification of Deltas in *A. queenslandica* and in other metazoan lineages (for example, *Lottia gigantea*, seven ligands; *Caenorhabditis elegans*, ten ligands) reflects a general theme in the evolvability of signaling pathway ligands [1]. The deeper relationships of Notch ligands generally are not well resolved, or do not reflect accepted animal phylogenies [15,18,21]. Due to their more recent shared ancestry, it is not unexpected that bilaterian ligands share features to the exclusion of the AmqDeltas. As well as the number and location of the EGF and DOS domains (Figures 1B, Additional file 2), essential binding sites identified in bilaterian DSL domains [22] are not conserved in the *A. queenslandica* ligands.

When considering the functional significance of *A. queenslandica* possessing five Delta ligands, and the possible interactions of these Deltas with the single Notch receptor, it is worth noting that Notch ligands are known to partake in diverse interactions outside of the canonical signaling context. For example, homotypic ligand-ligand interactions can play a role in cell adhesiveness [23], and ligands may be cleaved and released to act as soluble agonists [24] or antagonists [25] of signaling. Further, the Notch pathway can be negatively regulated via *cis* inhibition, in which ligands sequester receptor molecules intracellularly [26], or via the binding of dominant-negative forms of ligands that lack intracellular regions [27]. Although this latter effect was reported using engineered versions of truncated ligands, it is noteworthy that *AmqDelta1* similarly has a highly reduced intracellular domain (Figure 1B).

The role of multiple ligands may also be to facilitate the activation of reciprocal events in the signaling cell as a result of receptor/ligand binding. Such an effect could be exerted by the binding capacities of the ligand intracellular tails [28,29]. In bilaterians, the intracellular regions of DSL ligands are divergent and do not contain any globular domains, however they commonly possess lysine residues and a C-terminal PDZ domain binding motif [28]. These features enable interactions with PDZ-containing scaffold/adaptor proteins [30,31], as well as providing sites for ubiquitination and thus endocytotic regulation of ligands [32,33]. The intracellular tails of the *A. queenslandica* Deltas are similarly highly divergent, and are also predicted to contain multiple sites for ubiquitination as well as a variety of short linear motifs capable of binding to PDZ, PTB, SH2 and SH3 domains, amongst others (Figure 1B). This diversity may thus provide a variable interface for interactions between each ligand and the protein populations of signaling cells.

### Notch pathway expression in *A. queenslandica* development

**Figure 9:**
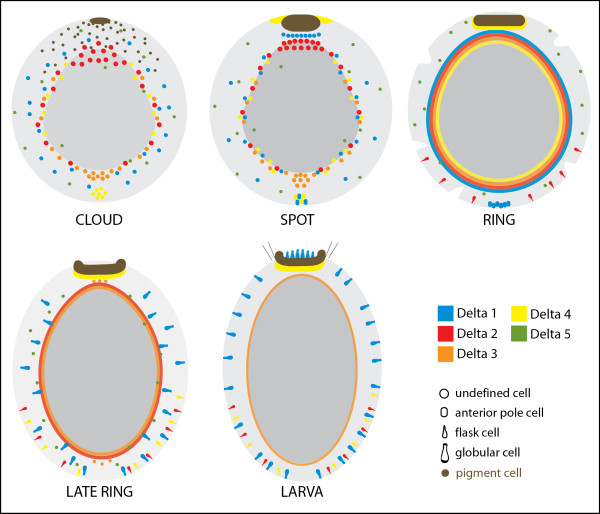
**Summary of the expression of *AmqDelta *genes during *Amphimedon queenslandica *development.** Schematic representation of *A. queenslandica* development highlighting the dynamic expression of *AmqDeltas* across multiple cell layers and cell types. Cell types are identified where possible; note that while each gene is represented with a unique territory of expression, coexpression of ligands in the same cell type is likely: for example, *AmqDelta1* and *AmqDelta4* in the anterior pole cells, spot stage.

The dynamic expression of the Notch receptor and ligands during the development of *A. queenslandica* is consistent with this signaling pathway contributing to a variety of embryonic processes. Regarding the timing of Notch pathway activation, it is notable that transcripts of the receptor or ligands generally are not detected during the early asymmetric cleavage events (white stage). Subsequently, multiple territories of Delta expression arise during the cloud stage, following the polarization of the embryo [34]. In later development, diverse cell lineages express Delta ligands (Figure 9), confirming that pleiotropic deployment of Notch signaling in *A. queenslandica*, as documented in the Bilateria and Cnidaria.

Three modes of activity are classically described for the Notch signaling pathway: lateral inhibition, boundary formation and asymmetric lineage decisions [3]. We find that the developmental expression patterns of Notch and its ligands in *A. queenslandica* are reminiscent of some of these classical Notch processes. In lateral inhibition, Notch signaling within equivalence groups singles out cells that will then follow a different developmental trajectory to the original population. Accordingly, we propose that the cell-type-restricted expression domains of the *A. queenslandica* Deltas may reflect Notch activity in shaping the development of these particular cell lineages to the exclusion of their neighboring cells (for example, *AmqDelta1*, anterior pole cells Figure 2D; *AmqDelta4*, flask cells Figure 5D). Regarding boundary formation, we propose that Notch signaling plays a role in determining the identity of the posterior ciliated cells that arise in a precise ring surrounding the pigment spot in *A. queenslandica*. In this region, the expression of *AmqNotch* and *AmqDelta4* are coincident with the morphological differentiation of the ciliated cells, which differentially express the cryptochrome-encoding gene *Aq-Cry2* (Figures 5B 7C and 8A) [35]. For the third mode of Notch activity, asymmetric lineage decisions, we are unable to determine whether there is asymmetric segregation of these molecules based on the expression of the ligands and receptor alone. The early divisions of blastomeres in *A. queenslandica* are certainly highly asymmetric [36], however we found no significant expression of Notch components during these stages.

### Homology of metazoan cell types based on Notch/Delta expression

Given the highly pleiotropic and context-dependent nature of Notch signaling, and the widespread co-option of signaling pathways into the generation of lineage-specific characters (see for example [37]), caution is required when proposing cell homologies between sponges and eumetazoans based solely on the localized enrichment of *Delta* and *Notch* transcripts. However, it is intriguing that many of the cells expressing Deltas in *A. queenslandica* may perform sensory functions in the larva, as a key role of Notch signaling in Eumetazoa is consistently in the specification, determination and differentiation of neural cell types [8,11,18,27,38-40]. *A. queenslandica* larval flask cells, which express *AmqDelta3* and *4*, are morphologically most akin to eumetazoan sensory cells, possessing a cilium that arises from a deep invagination in the cell and a basal nucleus that is surrounded by electron-lucent vesicles [20,41]. In contrast the globular cells, which express *AmqDelta1*, do not display conventional sensory morphology yet have previously been shown to express an ortholog of *atonal*, a basic helix-loop-helix (bHLH) neurogenic transcription factor [14], and components of the post-synaptic density [42]. The anterior pole cells (*AmqDelta1* and *4)*, also express a number of bHLH orthologs involved in neural development including *atonal* and *acheate-scute* (GSR and BMD, unpublished results) and due to their position, are likely to be involved in the settlement response of the larva [43]. The posterior ciliated cells (*AmqDelta4*), the only cells for which a sensory function has been explicitly confirmed [20,35,44], also express *acheate-scute* (Richards and Degnan unpublished), a homeobox gene with neural functionality (*LIM*) [45], and components of the WNT, TGFβ and Hedgehog signaling pathways [34,36,46]. That this suite of sponge larval cells expresses Delta ligands and other genes related to the development of neural cell types suggests a shared ancestry between non-neural sensory cells of poriferans and the neurons of the Eumetazoa.

The embryonic expression domains of several *A. queenslandica* Notch ligands persist into the larval period: for example, *AmqDelta1*, globular cells; *AmqDelta4*, posterior ciliated cells. This is in contrast to the transient ligand activity commonly reported in bilaterians, in which *Delta* expression occurs during the specification and/or differentiation of cell types, but then ceases once morphogenesis is complete [18,27,38,39,47]. In sponges, cellular plasticity, rather than ‘point-of-no-return differentiation’ [48], is a widespread feature, for example, larval epithelial cells dedifferentiate at metamorphosis and then redifferentiate into choanocytes in *A. queenslandica*[44]. The persistence of *Delta* signaling in some of the larval sponge cells may therefore be required to maintain cellular identity in lieu of these cells achieving a terminally differentiated state.

## Conclusions

Based on our own and other studies of the Notch pathway in *A. queenslandica*, we propose that the origin of this signaling mechanism can be minimally dated to the last common ancestor of the Demospongiae + Eumetazoa (this study, [1,14,15]). Whether this ancestor was also the metazoan LCA awaits resolution of branching orders at the base of the animal kingdom. Although we do not have functional confirmation of this hypothesis, the diverse structures of the *AmqDelta* ligands, and their coincident expression in the development of multiple cell types and regions in the larva, strongly suggests that Notch signaling plays an active role in *A. queenslandica* development.

The expression of Delta ligands in many *A. queenslandica* cell types before they have achieved their final morphologies and/or locations, leads us to propose that a conserved facet of Notch signaling in *A. queenslandica* is to mediate the choices made by non-terminally differentiated cells [6]. As such, Notch may be playing a developmental role by regulating the deployment of various cell differentiation gene batteries within the developing sponge embryo.

## Methods

### Sequence analysis

*AmqNotch* and *AmqDelta1* have been previously reported [14]. *AmqDelta2* to *5* were identified in the *A. queenslandica* trace archives ( http://blast.ncbi.nlm.nih.gov/Blast.cgi [Reniera_sp__jgi-2005_WGS]) by conducting tBLASTn searches using the conserved DSL domain of bilaterian Notch ligands. Traces recovered from these searches were assembled using an in-house assembly tool [34]. Primers were designed to each identified DSL domain and used in RACE reactions to obtain full-length coding sequences (BD Smart Kit, Clontech Laboratories, Mountain View, CA, U.S.A). cDNA templates for RACE were synthesized from RNA isolated from *A. queenslandica* developmental stages. (Genbank accession numbers: AmqNotch, EU273942; AmqDelta1, EU273941; AmqDelta2, GU385841; AmqDelta3, GU385842; AmqDelta4, GU385843; AmqDelta5, GU385844.)

Delta proteins from representative metazoan species were aligned to the conceptually translated *A. queenslandica* sequences using MUSCLE [49], and manually edited in SEAVIEW [50]. Conserved domain predictions were made using InterProScan (EBI); Delta EGF repeats and Delta/OSM-11 (DOS) motifs were manually annotated following the standards of Rasmussen *et al*. [18] and Komatsu [51] respectively.

For analysis of functional sites in the intracellular tails of AmqDelta protein sequences the ELM (Eukaryotic Linear Motif) server was used [19]. ELM results are filtered by species, but as sponges are not represented in the ELM organism menu, analyses were conducted using the *Homo sapiens* filter and then the *Drosophila melanogaster* filter and only sites retrieved by both analyses were retained.

### Whole mount *in situ* hybridization

Adult sponges were collected from Heron Island Reef (latitude: 23° 26′ 60 S, longitude: 151° 55′ 0 E, Great Barrier Reef, Australia) and developmental material was procured following [52]. Whole mount *in situ* hybridizations were carried out as described in [53] using digoxigenin-labeled RNA probes transcribed from PCR fragments that had been cloned into the pGemT vector (Promega, Madison, WI, U.S.A). Probe length and domain coverage: *AmqDelta1*, 900 bp, DSL + EGF; *AmqDelta2*, 760 bp, 5′ untranslated region (UTR) + DSL + EGF; *AmqDelta3*, 1.9 kb, DSL + EGF + TM; *AmqDelta4*, 960 bp, 5′UTR + DSL; *AmqDelta5*, 875 bp, DSL + EGF; *AmqNotch*, 3 kb, Notch/Lin repeats (NLR) + Ankyrin domains (ANK).

### Histology

Developmental stages were fixed and sectioned as described in [52]. Hemotoxylin and eosin staining of sectioned material was carried out as described in [54] with minor modifications. Images were captured using a Nikon Digital Sight DS-U1 camera (Nikon Australia Pty. Ltd. Lidcombe, Australia) mounted on an Olympus BX60F-3 compound microscope (Olympus Australia Pty. Ltd., Mt Waverly, Australia) with Nomarski optics. Adobe Photoshop CS2 (version 9.0.2) (Adobe Systems Inc., San Jose, CA, U.S.A.) was used to edit images for publication.

## Competing interests

The authors declare that they have no competing interests.

## Authors’ contributions

GSR performed the experiments. BMD and GSR conceived the study and drafted the manuscript. Both authors read and approved the final manuscript.

## Authors’ information

GSR: present address: Sars International Centre for Marine Molecular Biology, Bergen 5008, Norway.

## Supplementary Material

Additional file 1 **Delta alignment.** Sequence alignment used in Figure 1A. Click here for file

Additional file 2 **DOS domains in Delta proteins.** Sequence alignment of *A.queenslandica* Delta EGF repeats, analyzed for the presence of DOS domains. Click here for file
