# Supplementary material for: The expression of Delta ligands in the sponge Amphimedon queenslandica suggests an ancient role for Notch signaling in metazoan development
Source: EvoDevo. 2012 Jul 23;3:15. doi: 10.1186/2041-9139-3-15 (PMC3482393; doi:10.1186/2041-9139-3-15)
Supplement: Additional file 1 — Delta alignment. Sequence alignment used in Figure 1A. [file 2041-9139-3-15-S1.rtf]

Additional file 1: Delta alignment


Delta1  : MINAFICLL--FVLVSCTQFVHC--DYTAEIRIFSYGNPRKIHRSIGAGNSLTYCCCDCQGQPTCCNSGAAQS---CAHNCNTKVL :  79
Delta2  : -MYTLDAVA--FFLLGLLLH-QVNGDYELLFRFRRYSNPT-----------------DRDNNDNCCDNLLI-----CVSHCDTLFR :  60
Delta3  : MNGWLL-----LPCLSLLSH-LCTGRYTLLVKAVQYHNPD-----------------NQDWNDGCCDWP-------CSNNCDLYFK :  56
Delta4  : ------MMI--ILLLMCLIEPLKASVFTLKLEFHNYDNPN-----------------GWNSVKVCCDRGPLSGT--CYSPCETKFS :  59
Delta5  : -MKIFQALV-----ISVSLLWNATGDFTLSFQFHRYSNPS-----------------DRDVNGNCCDTAPGVI---C-GSCDTHFK :  59
DmDelta : -MHWIKCLLTAFICFTVIVQVHSSGSFELRLKY--FSNDH-----------------GRDNEGRCCSGESDGATGKCLGSCKTRFR :  66
 
                                                                                                                                                                                                      
Delta1  : I-CIADK--------YCVTST----------YMAQGNQTFTNTAFGSLANPIYINVPGALPFQ-VKQLFQFFLYR---SADSMP-- : 140
Delta2  : SLCLRNRDSNSEP--QCIPGT-VYDPGTVGDYNGPDNSITFGESVGSISNPIIYERPGSIPESGFQLYMEVFDYD---SRNDND-- : 138
Delta3  : F-CIRNKGLSASNINNCWDSVQTYGDVSTNNYYFPNYGELYPG--ARIWNDLTFNRNEPWPGS-VQVLVESLDAD----DNADD-- : 132
Delta4  : I-CLRNGNTSQID-KSCPSSSDIMTSGIV-----PGDSLTFGASIGSLPNPLTF-YLDSLHFSGVQVYIKIEDDDLGIYDDDLNQD : 137
Delta5  : SLCLRNGGTSHSQTGQCIPGT-VLSPGGVG-----GSSVNFSAHIGVISNPFGYNKSGNILQSGFQLYLEVWDDD---YAFNDD-- : 134
DmDelta : V-CLKHYQATIDTTSQCTYGD-VITPILGENSVNLTDAQRFQN--KGFTNPIQFPFSFSWPGT-FSLIVEAWHDT---NNSGNART : 144
                                                                                                   
                                                                                                 
Delta1  : ---LIKE--FTENAAVRLDIMREFF--NEENFVSLHYNITYIC-SNDYYGTTCSLYCKAYNDSTNGHYTCNS-AGQKTCLAGYTNT : 217
Delta2  : ---YIDSIVLNIPAQPTTERQSTVV--GEDGKISLELSYSLSC-SQNYYGSDCSQQCIPRNDNTNGHYTCNTTTGGIICREGWQNI : 218
Delta3  : ---LIDRNAFNLDLSPNGQWSNELYANGYYDRAQFKIRVRLFC-QQNYYGSNCNVYCVQQNDDTNGHYTCGS-DGAKICNNGYTNP : 213
Delta4  : ITDLIDELFVQIPSNGRNAAYRMMY--GIHGVASINASYQLACTDDNYYGNDCSVYCKPQNDESAGHYSCNQTTGEKICLSGYVDP : 221
Delta5  : ---LIDRIVFDILNRTSSQISHTAI--GVFNRVSLLSSYRLLC-SVNYYGFDCSVLCIPYNDDTNGHYTCNSTTGAKKCREGWQNV : 214
DmDelta : NKLLIQRLLVQQVLEVSSEWKTNK---SESQYTSLEYDFRVTC-DLNYYGSGCAKFCRPR-DDSFGHSTCSE-TGEIICLTGWQG- : 223
    
                                                                                                                                                                                                    
Delta1  : SNNCLERIIVCREGCHPTGGYCTVSNQCLCNNGWTGTNCSISTTCSSC--TNGVCYEPNECTCNHGWIGLDCSTP--VCDPPCSG- : 298
Delta2  : TTNCT--DVACHLVCQSPGGNCSSDGTCECNSNWFGDQCQNPNCTEGCHPEGGFCNMPNECLCHSNWNGTLCDEL--LCNVNCSS- : 299
Delta3  : SGNCL--TPICSSGCSSQNGYCNVPGECLCNTGWTGTNCNECIPKSGCSTSHGYCNVANECLCETGYGGSLCTEDHDVCGHEAPCL : 297
Delta4  : SSNCT-------------------------------------------------------------------------CTVNNPN- : 233
Delta5  : TNNCT--NVACNLVCRSPGGTCTHNGTCQCTIGWSGDGCDIPQCIEGCHPQGGYCTEPYQCLCYNNWNGSLCNES--FCTVNCSS- : 295
DmDelta : -DYCH--IPKCAKGCEH--GHCDKPNQCVCQLGWKGALCNECVLEPNC--IHGTCNKPWTCICNEGWGGLYCNQDLNYCTNHRPCK : 302

                                                                                                       
                                                                                            
Delta1  : ---STNIAMYVSIINVIINIIIVLLLIGICALY-FTRKTRQ---------------------YDLKGSELTKI------------- : 532
Delta2  : -----VTAIIGSVIGAIMLIIIVVISLFIVILL-KKRRQGL----------------KNRSKSSVVNYNLVKGSNDQLSLDHNNNY : 561
Delta3  : -NGGVGMLVGVSVGGALLSCCFAMIIVGCCCYL-CRKKTQMKKFEIPIEGSHFSDNPTYIPPELLQSTTLDHSASPQYGTSAQKLY : 736
Delta4  : -------VIIAAVITAFVLIGVITIFVSVIILL-LWKRKQK-----------------PLPDIDKTGTIYDSTAKESSISGNNNLE : 368
Delta5  : -------IVGGIVGSLFLTILLITAFVAVSVIASCFKKRKM-----------------RNHKEIHTSDAMDNVA------------ : 559
DmDelta : ARADGLTNAQVVLIAVFSVAMPLVAVIAACVVF-CMKRKRKRAQEKD------DAEARKQNEQNAVATMHHNGSGVGVALASASLG : 663
