# Supplementary material for: The expression of Delta ligands in the sponge Amphimedon queenslandica suggests an ancient role for Notch signaling in metazoan development
Source: EvoDevo. 2012 Jul 23;3:15. doi: 10.1186/2041-9139-3-15 (PMC3482393; doi:10.1186/2041-9139-3-15)
Supplement: Additional file 2 — DOS domains in Delta proteins. Sequence alignment of A.queenslandica Delta EGF repeats, analyzed for the presence of DOS domains. [file 2041-9139-3-15-S2.doc]

**Additional file 2: DOS domains in Delta proteins**

An alignment of the first 2 EGF repeats and/or DOS region of bilaterian proteins with all EGF repeats of the *A. queenslandica* ligands (Amq EGF pairs are numbered 1-2; 2-3 *etc.*). Diagnostic residues of the bilaterian EGFs 1-2 and DOS are represented under the alignment. The *A. queenslandica* proteins do not have the characteristic basic “**B**” residue between cysteines “C” 4 and 5 of the first repeat. Some of the *A. queenslandica* proteins have the proline “P” between the cysteines 2 and 3 of the second repeat, but none also have the tryptophan “W” residue prior to the fourth cysteine of the second EGF (although some have other hydrophobic residues *e.g.* tyrosine, Y). Dashes indicate gaps; residues are shaded according to the level of conservation at each position. 100%, black; 80%, dark grey; 60%, light grey. Amq, *Amphimedon queenslandica*; Dm, *Drosophila melanogaster*; Hs, *Homo sapiens*; Ce, *Caenorhabditis elegans*. (note: *C. elegans* OSM and DOS proteins only display conserved DOS residues, not the conserved EGF1-2 organisation, as they are not conventional Delta/Serrate/Jagged type ligands).
